# Large-scale suppression of recombination predates genomic rearrangements in *Neurospora tetrasperma*

**DOI:** 10.1038/s41467-017-01317-6

**Published:** 2017-10-26

**Authors:** Yu Sun, Jesper Svedberg, Markus Hiltunen, Pádraic Corcoran, Hanna Johannesson

**Affiliations:** 1 Guangdong Provincial Key Laboratory of Protein Function and Regulation in Agricultural Organisms, College of Life Sciences, South China Agricultural University, Guangzhou, 510642 China; 2 Department of Organismal Biology, Uppsala University, Norbyvägen 18D, 752 36 Uppsala, Sweden; 3 Department of Animal and Plant Sciences, University of Sheffield, Sheffield, S10 2TN UK

## Abstract

A common feature of eukaryote genomes is large chromosomal regions where recombination is absent or strongly reduced, but the factors that cause this reduction are not well understood. Genomic rearrangements have often been implicated, but they may also be a consequence of recombination suppression rather than a cause. In this study, we generate eight high-quality genomic data sets of the filamentous ascomycete *Neurospora tetrasperma*, a fungus that lacks recombination over most of its largest chromosome. The genomes surprisingly reveal collinearity of the non-recombining regions and although large inversions are enriched in these regions, we conclude these inversions to be derived and not the cause of the suppression. To our knowledge, this is the first time that non-recombining, genic regions as large as 86% of a full chromosome (or 8 Mbp), are shown to be collinear. These findings are of significant interest for our understanding of the evolution of sex chromosomes and other supergene complexes.

## Abstract (web summary)

Genomic rearrangements can be either a cause or a consequence of large-scale suppression of recombination in a genome. Here, Sun et al. show that recombination may be suppressed in collinear genomic regions, and that inversions may accumulate over time in a derived manner.

## Introduction

Meiotic recombination plays an important role in maintaining and creating variation in sexual organisms, but can also break up advantageous gene complexes and thereby act as a barrier against establishing complex phenotypes. Suppression of recombination can in the latter case evolve to ensure that gene complexes are inherited together^1^. Complex phenotypes that segregate as Mendelian traits have often been mapped to multi-gene regions where recombination is reduced. Theory predicts that mutations that reduce recombination between genes in advantageous gene complexes will be favoured^2^, leading to the formation of so-called ‘supergenes’^1,3^. Numerous supergenes have recently been identified, for instance linking together genes determining phenotypically distinct morphs of mimetic butterflies^4^ and ruffs^5,6^, social structures in fire ants^7^ and locally adapted ecotypes of monkeyflower^8^. In the most well-studied supergene systems, those of animal and plant sex chromosomes, sexually antagonistic selection is thought to have driven an expansion of a region of suppressed recombination along almost the entire chromosome carrying the sex determination locus^9^.

In spite of the importance of recombination suppression for the evolution of adaptive linkage, it has been difficult to determine the underlying mechanisms for reducing recombination. Besides heterochromatin, which is generally restricted to repetitive regions of the chromosomes, all known examples of large-scale suppression of recombination are associated with structural rearrangements. Chromosomal inversions will, as single mutational events, immediately affect recombination patterns within the inverted region of the chromosome and are often proposed as causative mechanisms for recombination suppression^10,11^. In many supergene systems, including several young sex chromosomes^12,13^, inversions have been found in the regions of suppressed recombination, but on older sex chromosomes the degeneration of the heterogametic sex chromosome has precluded any direct identification of inversions. In humans, so-called evolutionary strata (regions with distinct levels of sequence divergence) have instead been identified, and these have been interpreted as being caused by a succession of structural rearrangements such as inversions and chromosomal fusions^14^. However, the strata hypothesis is not supported by all empirical data. Specifically, in sex chromosomes of humans^15^, *Silene latifolia* ^16^ and threespine stickleback^17^ there are regions where a gradual change in divergence levels has been interpreted as the result of a gradual expansion of suppressed recombination, but these regions are still associated with putatively rearranged regions or could be affected by mechanisms that scrambles the signal of divergence, such as gene conversion^18^. The ancient and highly diverged nature of many sex chromosome systems and the technical issues of studying structural variation in large genomes have so far made it difficult to conclusively untangle the relationship between suppression of recombination and structural rearrangements.

A further complicating factor is that while inversions can be advantageous by reducing recombination, they may also be associated with fitness costs. Crossover events within an inversion often cause unbalanced, inviable, gametes and can also have negative effects of fertility that increase with the size of the inversion^19, 20^. If another, non-structural mechanism suppresses recombination, selection against inversions would be relaxed and potentially increase their frequency in a population. Thus, an inversion found in a region of low recombination could instead be a consequence of suppressed recombination, rather than the cause.

Analogous to sex chromosomes, suppression of recombination has been identified along mating-type (*mat*) chromosomes of certain fungi^21–23^, including the filamentous ascomycete *Neurospora tetrasperma*. This species is a close relative to the model fungus *N. crassa* and maintains a self-fertile, pseudohomothallic, life cycle by producing ascospores that contain both necessary mating types in two different haploid nuclei. The ascospores can then germinate to form a heterokaryotic mycelium which can produce new sexual spores by selfing^24^. At meiosis, recombination suppression between the *mat* locus and the centromere ensures the proper packaging of the nuclei of opposite mating types into an ascospore^25^. However, recombination is suppressed far beyond this region and is lacking in most of the chromosome. Recombination suppression in *N. tetrasperma* is evidenced from analysis of marker segregation in crosses^26^ and cytological examinations have shown a lack of synapsis of most of the *mat* chromosome at pachytene^26^. Population genetic analyses have also shown that sequence divergence is elevated between the *mat* chromosomes, and that linkage disequilibrium is extensive^27–29^.

Ellison et al.^30^ generated high-quality assemblies from the two haploid genomes of one heterokaryotic strain of *N. tetrasperma*. These assemblies showed that one of the *mat* chromosomes carries several overlapping inversions covering much of the region where recombination is suppressed, whereas the *mat* chromosome of the other nuclei is collinear with *N. crassa* ^31^. The interpretation from this analysis was that the inversions were central in the evolution of the pseudohomothallic mating system of *N. tetrasperma*, and the cause of the recombination suppression. However, the influence of both structural rearrangements and unlinked recombination modifiers of recombination suppression has also been demonstrated experimentally in the same strain^32^.

*Neurospora tetrasperma* shows strong population structure and has been divided into at least eight phylogenetically distinct lineages^27,33^. All lineages show elevated divergences on the *mat* chromosome, but the size of the region and level of divergence varies between them^27^. If the inversions identified by Ellison et al.^30^ were fixed in the lineages, this would support the model that they represent an ancient and central event in the evolution of *N. tetrasperma*. Here, we investigate the structural variation on the *mat* chromosome to determine whether the previously identified inversions are shared between lineages and thus, can be interpreted as an ancestral trait of the species. We sequence eight haploid *N. tetrasperma* genomes from four heterokaryons, originating from four different lineages, and use these data to investigate their chromosome structure. Taken together, our results show that the majority of *N. tetrasperma* strains are collinear. All newly discovered inversions show limited phylogenetic distribution, indicating that they have taken place after the establishment of the non-recombining region. Collinearity is therefore the ancestral state of *N. tetrasperma* and all inversions that have been identified by Ellison et al.^30^ and herein are derived. These results establish the existence of large-scale recombination suppression in the absence of structural rearrangements, and caution against interpreting the presence of such rearrangements as being causative.

## Results

### A high degree of collinearity among *Neurospora* genomes

**Fig. 1:**
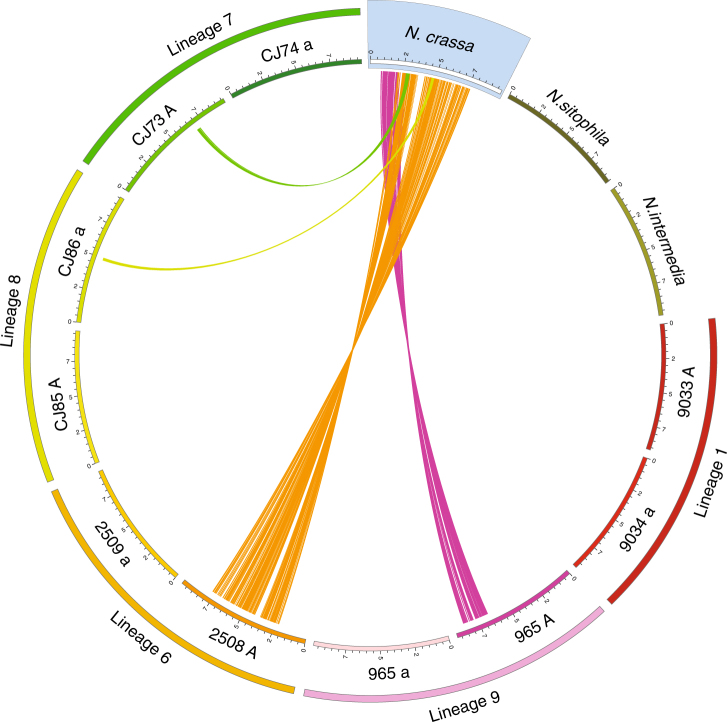
Whole chromosome pairwise alignments of the *mat* chromosome of *N. tetrasperma* against *N. crassa*. The *mat* chromosome (chromosome 1) of the 10 high-quality de novo assemblies (eight *N. tetrasperma*, one *N. sitophila* and one *N. intermedia*) generated for this study (Table 1), together with two *mat* chromosomes from Lineage 6^30^, were aligned to the *N. crassa* OR74A assembly^31^. Chromosomes are scaled in Mbp and only inverted regions larger than 100 kbp are shown. For *N. tetrasperma*, genomes of each of the two mating types from a single heterokaryon were sequenced separately. Each heterokaryon represent a phylogenetically distinct lineage (Lineages 1, 6, 7, 8 and 9)^27,33,34^. The previously identified set of inversions in 2508 *mat A* (Lineage 6)^30^ is the biggest inverted region identified in *N. tetrasperma*. Strain 965 *mat A* (Lineage 9) carries a 1.9 Mbp inversion surrounding the *mat* locus, CJ86 *mat a* (Lineage 8) carries a 260 kbp inversion and CJ73 *mat A* (Lineage 7) a 320 kbp inversion. Both mating type strains of Lineage 1 completely lack large inversions on this chromosome, as do the *N. sitophila* and *N. intermedia* genomes

For three of the eight investigated *N. tetrasperma* genomes (Table 1), we created high-quality assemblies using optical mapping data together with Illumina paired-end and mate-pair reads, and for the other five we generated PacBio long-read data. Using these data sets, we were in all eight cases able to assemble the seven expected chromosomes into complete, or near-complete scaffolds and contigs (Table 1, Supplementary Tables 1 and 2, Supplementary Fig. 1). Whole-genome alignments against the *N. crassa* and *N. tetrasperma* reference genomes showed that all of these eight assemblies were mostly collinear with *N. crassa* and *N. tetrasperma mat a*, and none carried the inversions identified in *N. tetrasperma mat A* by Ellison et al.^30^ (Fig. 1, Supplementary Fig. 1). Furthermore, in order to expand our knowledge on the conservation of collinearity among the terminal, conidiating group of *Neurospora*, we sequenced two closely related species^34^ using PacBio: *N. sitophila* and *N. intermedia* (Table 1, Supplementary Table 2). Both genomes show a high degree of collinearity with both *N. crassa* and *N. tetrasperma*, indicating strong conservation of gene order in this clade (Supplementary Fig. 1).

**Table 1:** Strains sequenced for this study

Species	Homokaryon ID	Mating type	Heterokaryon ID	Lineage	Sequencing technology
*N. tetrasperma*	9033	*mat A*	P4492	L1	Optical mapping, Illumina
	9034	*mat a*			Optical mapping, Illumina
	CJ73	*mat A*	J2	L7	PacBio
	CJ74	*mat a*			PacBio
	CJ85	*mat A*	P505	L8	PacBio
	CJ86	*mat a*			PacBio
	965A	*mat A*	965	L9	Optical mapping, Illumina
	965a	*mat a*			PacBio
*N. intermedia*	8807	*mat a*			PacBio
*N. sitophila*	W1434	*mat A*			PacBio

### Lineage-specific inversions on the *mat* chromosome

In spite of general collinearity among the *Neurospora* genomes, several large inversions were found in *N. tetrasperma*. Using whole chromosome alignments, we detected a previously unknown 1.9 Mbp inversion surrounding the *mat* locus and two other large inversions (260 and 320 kbp) on the *mat* chromosome of *N. tetrasperma* (Fig. 1). In addition to our high-quality genomic data sets, we also analysed a published set of 92 *N. tetrasperma* strains sequenced with Illumina paired end reads^27^ (Supplementary Table 3). By aligning fragmented de novo assemblies of these 92 strains against both the *N. tetrasperma mat a* and *mat A* assemblies, we verified both breakpoints of one of the three inversions detected by Optical mapping or PacBio in Lineages 7, 8 and 9 (Fig. 1), while for two inversions we were only able to verify one breakpoint due to highly repetitive regions surrounding the second (Supplementary Table 4). Using the Illumina-data, we also identified three new unique inverted regions larger than100 kbp on the *mat* chromosome of Lineages 4 and 10, and show that the inversions identified by Ellison et al.^30^ on *mat A* in Lineage 6 are fixed on all *mat A* chromosomes in the sister clade Lineage 5 (Supplementary Table 4). All inverted regions over 100 kbp could further be shown to be fixed within one mating type in each lineage, except for a 3.6 Mbp inversion found in a single strain of Lineage 10 (CJ01) and a cluster of overlapping inversions (400 kbp in total) found on chromosome 5 in four genomes of Lineage 10 (Supplementary Table 4). For all inversions larger than 100 kbp that were identified in the Illumina data but not confirmed with other sequencing data, we used PCR, and in most cases also Sanger sequencing, to verify the breakpoints (Supplementary Table 4, Supplementary Fig. 2).

### Enrichment of large inversions

A mechanism causing suppression of recombination is expected reduce the negative fitness effects of inversions in these regions. Large inversions that would have large fitness effects in recombining regions would then be expected to accumulate in regions where recombination is suppressed^19^. For all inversions (>1 kbp, identified in at least two different strains, Supplementary Table 5), we found an even distribution between the region of suppressed recombination and the rest of the genome (1.08×10^−6^ inversions per base pair for the region of suppressed recombination vs. 1.39×10^−6^ inversions per base pair for the rest of the genome). However, the mean size of inverted regions in the region of suppressed recombination is 45 times larger than in the rest of the genome (1.5 vs. 0.033 Mbp) and when including only the inverted regions larger than 100 kbp (Supplementary Table 4) in the analysis, we found a 22-fold enrichment per base pair in the region of suppressed recombination compared to the rest of the genome (*p* = 0.00061, one-sided binomial test). These results suggest that large inversions are significantly enriched in the suppressed recombination region in *N. tetrasperma*. When expanding the analysis by performing interspecific comparison between *N. intermedia*, *N. sitophila* and *N. crassa* only one inversion larger than 100 kbp is found in *N. sitophila* (Supplementary Fig. 1), which shows that inversions in *Neurospora* genomes are rare and further indicates a selection against large inversions in *Neurospora* species, but that the selective strength is reduced in the region of suppressed recombination on the *mat* chromosome in *N. tetrasperma*.

## Discussion

**Fig. 2:**
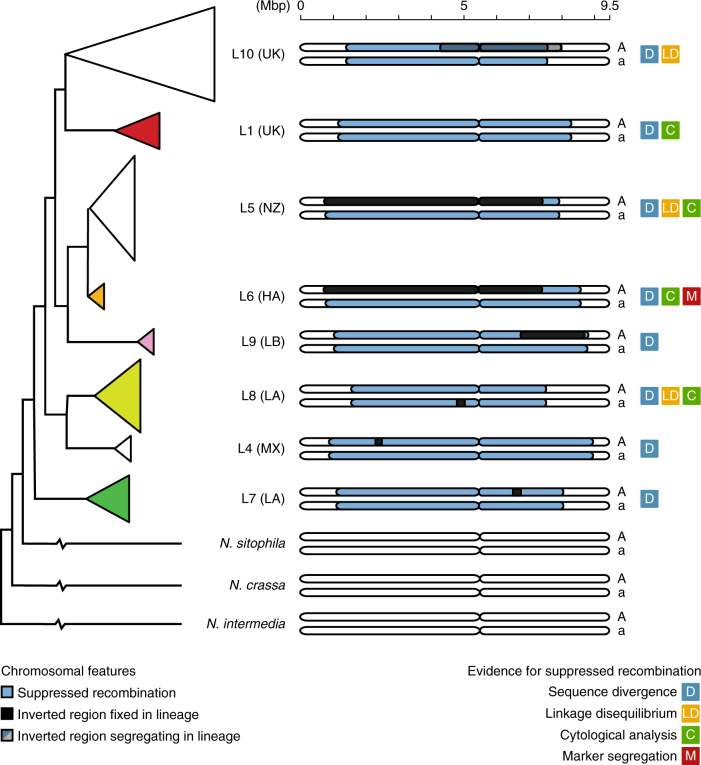
Structure of the *mat* chromosome across *N. tetrasperma* lineages. All inversions above 100 kbp on *mat* chromosomes identified in this study, plotted per lineage. The phylogenetic tree is adapted from Corcoran et al.^27^ and the colours of the filled triangles correspond to the colours used in Fig. 1. Several studies have provided different types of evidence for suppression of recombination along the *mat* chromosome in *N. tetrasperma*. Gallegos et al.^26^ investigated strains from Lineages 1, 5, 6 and 8 and determined both that they lack chromosomal synapsis at pachytene in most of the *mat* chromosome, and that no crossovers occurred between a set of genetic markers for a Lineage 6 heterokaryon. Corcoran et al.^27^ identified a region of elevated sequence divergence between the mating types within a heterokaryon for all *N. tetrasperma* lineages included in this study, as well as a region of elevated linkage disequlibirium for three of them. The combination of evidence indicates that all *N. tetrasperma* lineages carry a region of suppressed recombination on the *mat* chromosome (blue bars), but the size of this region differs between lineages^27^. Black bars show inverted regions that are fixed on a mating type within a lineage. All identified inverted regions are fixed in all investigated strains of the same mating type within its lineage, except a 3.6 Mbp inversion only identified in CJ01 *mat* A in Lineage 10. The inverted region in Lineages 5 and 6 is the only one that is shared between lineages. *Neurospora sitophila*, *N. crassa* and *N. intermedia* share the gene order with the majority of *N. tetrasperma* strains, but the *mat* chromosome recombines freely. This distribution of inversions shows that all identified inversions are derived and that only the suppression of recombination is shared between all analyzed strains of *N. tetrasperma*

Previous studies have, through a number of different strategies^26–29^, established that the *mat* chromosome of *N. tetrasperma* contains a region where recombination is suppressed between the two mating types (Fig. 2), and based on an earlier genomic study^30^ inversions were hypothesized to be the causative factor. Here we show that the majority of *N. tetrasperma* lineages do not carry inversions capable of causing the large-scale suppression of recombination on the *mat* chromosome (Fig. 2). The inversions identified by Ellison et al.^30^ are limited to the *mat A* chromosome in Lineages 5 and 6, and while several lineages carry smaller inversions (2 Mbp or less) on one mating type chromosome, both *mat* chromosomes of Lineage 1 are completely collinear. It is therefore clear that something else than structural heterozygosity must maintain the suppression of recombination, covering a region as large as 8 Mbp or ~86 % of the entire chromosome^27^.

The phylogenetic distribution of inversions (Fig. 2) also supports that the ancestral state of the non-recombining region must have been collinear and that the detected inversions are derived. We have previously reported on introgression of *mat* chromosomes in several lineages of *N. tetrasperma* from other species of *Neurospora* for which recombination is not suppressed^27^. This phenomenon complicates the interpretation of our data, in that introgression could have potentially reestablished collinearity from an ancestrally inverted stage. However, several lines of evidence speak against this hypothesis. Firstly, Lineages 1 and 8, which are generally collinear, do not show any signs of introgression in either mating type^27^. Secondly, the inverted region of Lineages 5 and 6 *mat A* is located on a chromosome that displays a strong signal of introgression from an unknown *Neurospora* species, which makes it unlikely that it represents the ancestral gene order. Thirdly, while *mat A* of Lineage 9 shows signs of introgression, it is *mat a* that carries the 2 Mbp inversion detected in this lineage.

It is worth pointing out that it is difficult to rule out that the inversions we see in this study took place in the genomic background of other species of *Neurospora* and were subsequently brought into *N. tetrasperma* by introgression. This scenario does not question that recombination suppression evolved in collinear chromosomal regions, but would lead to an overestimation of inversions that have taken place in the region of suppressed recombination in *N. tetrasperma*. This concern is only relevant for the inversions in Lineages 5 and 6, since for the other lineages it is the non-introgressed chromosome^27^ that carries the inversion. The fact that the gene order is highly conserved between all species of *Neurospora* included in this study, and the *mat* chromosome of *N. tetrasperma* sticks out by breaking this pattern, suggests that the conserved gene order is the ancestral, and that changes have taken place in *N. tetrasperma*.

We do not yet know what causes the suppression of recombination in *N. tetrasperma*, but there are several possible mechanisms. Sequence divergence has been shown to reduce recombination^35^, but since recombination is restored when introgressed^32^ into the more diverged *N. crassa* ^36^, this is an unlikely mechanism for suppression in this system. Heterochromatin reduces recombination^37^, but there is no evidence that the *mat* chromosome would be heterochromatinized in *N. tetrasperma*. Constitutive heterochromatin is limited to low-GC, repetitive regions^38^ and facultative heterochromatin is not accumulated in the non-recombining region during vegetative growth^39^. Recombination hotspots could be depleted in the non-recombining region, but since this region shows signs of large-scale introgression in several lineages of *N. tetrasperma* ^27,36^, a stable depletion of hotspots is also unlikely. Jacobson showed that the genetic background affects recombination on the *mat* chromosome^32^ and genes that affect recombination rates locally have been found in *Neurospora* ^40^, but there are no known genes that can affect recombination over as large regions as detected in this study. In yeast^41^ and mammals^42^ recombination hotspots are enriched for H3K4me2 histone methylation^43^, and in Arabidopsis^44^ and Ascobolus^45^ DNA methylation is also known to affect recombination rates. The presence or absence of similar modifications throughout the non-recombining region could potentially affect double-strand break formation and recombination initiation in *N. tetrasperma*. Little is known about the fine-scale recombination landscape and what shapes it in *Neurospora* species, and other ascomycetes show variation in both large-scale and small-scale distribution of crossovers, as well as the regulation of this distribution^46–49^, which makes definite predictions difficult. Further data on, for example, the distribution of crossovers, double-strand breaks and chromatin modifications would be necessary to understand recombination in *Neurospora* and for identifying the underlying causes of recombination suppression.

Our results show that large-scale suppression of recombination in genic regions can remain over evolutionary time, even in absence of structural rearrangements. We also show that the region of suppressed recombination has an accumulation of large derived inversions, supporting the hypothesis of relaxation of selection against inversions in regions of suppressed recombination. These results stress the point that the association of inversions with low levels of recombination is not enough to infer that the inversion is the cause of the suppression. Furthermore, a similar mechanism for suppressing recombination could potentially also explain the strata seen on the human Y chromosome. To what extent recombination suppression by non-structural means exist in other species is still unknown, but regulation of recombination rates show both conservation and divergence between as distantly related organisms as plants, fungi and animals^50^. The advent of improved methods for whole-genome sequencing should make it easier to determine how widespread structural rearrangements are in regions of suppressed recombination across species, and if large-scale recombination suppression in collinear regions is a common phenomenon.

## Methods

### Strains investigated in the study

We used three publicly available, well-annotated *Neurospora* genomes in this study, including *N. crassa* (*N. crassa* OR74A version 12^31^, sequenced by Broad Institute, corrected for the assembly error detected by Galazka et al.^51^), *N. tetrasperma* FGSC 2508 *mat A* and FGSC 2509 *mat a* (both sequenced by JGI^30^). In addition, 92 previously published *N. tetrasperma* genomes assembled from Illumina paired-end data^27^ were analysed.

For this study, we gathered genomic data from eight haploid strains of *N. tetrasperma*, originating from four heterokaryotic strains. These heterokaryons represent phylogenetically and reproductively isolated lineages of the monophyletic morphospecies^27,33,34^, and were obtained in previous studies by isolating mycelia grown from asexual spores (conidia) of single mating-type that the fungus occasionally produces^52^. Finally, we gathered genomic data from one *N. sitophila* and one *N. intermedia* strain (Table 1). All strains sequenced for this project were obtained from the Fungal Genetics Stock Center^53^ (http://www.fgsc.net/) except *N. sitophila* W1434, which was provided by Jacobson et al.^54^.

### Illumina short-read data and optical mapping

We assembled high-quality genomes of three *N. tetrasperma* strains, using a combination of Illumina short-read data and optical mapping (Supplementary Table 1). We generated paired-end Illumina libraries with 500 bp insert size of these strains for an earlier study^27^ and these data were supplemented in this study with reads from 5 kbp mate-pair libraries (Supplementary Table 1). Strains were inoculated on a Vogel medium plate with 1% sucrose at 25 °C and DNA was extracted using the Easy-DNA Kit (Invitrogen) and sent to the Beijing Genomic Institute (BGI, Shenzheng, China) for sequencing. The DNA was fragmented, circularized and sequenced using Illumina HiSeq, generating 90 bp mate-pair reads. A series of filtration steps were set to remove the low-quality reads, including (1) 10 bp of the read for 500 bp library and 5 bp of the read from both ends for 5 kbp library, (2) reads with >10% of Ns’, (3) reads with >40 low-quality base calls (<Q_20_), (4) adapter contamination, and duplications. We used SOAPdenovo^55^ (http://soap.genomics.org.cn/soapdenovo.html; version 1.05) for the de novo assembly of the sequenced genomes. By testing multiple kmers, we chose kmer 21 as the final optimal value for de novo assembly. Single base correction and gap filling were performed by SOAPaligner (http://soap.genomics.org.cn/soapaligner.html; version 2.21).

To generate optical maps for the three strains, the fungal mycelium was digested by the lysing enzyme at 37° for 3 h. The collected DNA and protoplasts were centrifuged, and washed twice by phosphase buffer, and then suspended in STC buffer (0.125 M EDTA, pH 7.5, 0.9 M sorbitol). Equal volume of 2% low melting point agarose (LMPA) was poured into the medium to make the DNA plug. Argus^TM^ Optical Mapping System (OpGen) was used to evaluate the quality of DNA, and samples with a DNA molecule length of >150 kbp were considered as adequate quality for the optimal restriction enzyme digestion. The restriction enzymes were chosen by the OpGen Manager software, DNA samples were digested by MapCard (OpGen) and OpGen enzyme kits and the Argus^TM^ Optical Mapping System was used to collect the data. The restriction map was assembled into an optical map using OpGen optical map assembly, and the contigs generated by SOAPdenovo were placed and scaffolded using the OpGen alignment tool. Sequencing information, assembly statistics and data availability is shown in Supplementary Table 1.

### Genome sequencing and assembly using PacBio

Whole genomes of five *N. tetrasperma*, one *N. intermedia* and one *N. sitophila* strain (Supplementary Table 2) were sequenced using the PacBio RSII platform^56^ (Pacific Biosciences). The strains were cultured by adding conidia to 200 ml of 3% liquid malt extract medium in 500 ml Erlenmeyer flasks, which were placed on rotary shaker at 30 °C for 3–4 days. The cultures were harvested by removing the mycelium from the liquid, placing it on a filter paper, which was folded between several layers of tissue paper and pressed to remove excess liquid. The mycelium was cut into small pieces and ~1 g was allotted into 2 ml tubes with screw-on caps, after which the tubes were stored at −20 °C.

To extract the DNA, two tubes of each strain were freeze-dried overnight and macerated using a TissueLyzer II bead-beater (Qiagen). Two 2 mm metal beads were placed in each tube, which were then shaken for 20 s at 25 Hz. If a tube contained large pieces of freeze-dried mycelium, it was shaken for another 10 s. This procedure was repeated until no large pieces remained, but no tube was shaken for more than 40 s in total. DNA was extracted from the macerated samples using Genomic Tip G-500 columns (Qiagen) and cleaned using the PowerClean DNA Clean-Up kit (MoBio Labs).

The extracted DNA was sent to the Uppsala Genome Center (Science for Life Laboratory, Uppsala, Sweden), where libraries were prepared and sequenced on a PacBio RSII system, using four SMRT cells per sample and the C4 chemistry and P6 polymerase (Pacific Biosciences). The raw sequence data was then filtered and assembled using the SMRT Analysis package and HGAP 3.0 assembler^57^ (Pacific Biosciences, https://github.com/PacificBiosciences/). Sequencing information, assembly statistics and data availability is shown in Supplementary Table 2.

### Genomic comparison for the high-quality *Neurospora* genomes

The genomic comparison for all complete *Neurospora* genomes (three publicly available, three optical mapping and seven PacBio) were generated by LASTZ^58^ (http://www.bx.psu.edu/miller_lab/dist/README.lastz-1.02.00/README.lastz-1.02.00a.html; version 1.02) with low sensitive setting parameters, including no transition, step = 20 for seeding and no gap extension during alignment. The comparative genomics results were visualized by Circos^59^ (http://circos.ca/; version 0.64) in Fig. 1. Only inverted regions that were larger than 100 kbp, or located within a larger inverted region, were shown in the figure. The alignments for the inverted region were also manually inspected to control for alignment errors.

### Identifying inversions from fragmented de novo assemblies

The de novo assemblies of 92 *N. tetrasperma* strains that had previously been generated from short paired-end Illumina reads^27^ were examined for the presence of inversions. These de novo assemblies were highly fragmented, with scaffold numbers ranging from 242 to 34,302 (mean 2845) and N50 from 23,658 to 1,140,656 bp (mean 172,026 bp) (Supplementary Table 3). In order to identify inversions, the MUMmer^60^ whole-genome aligner was used to align the 92 assemblies to the high-quality assemblies of the *N. tetrasperma* 2508A and 2509a strains (parameters: nucmer –c 200 –b 2000). Candidate inversions were called using custom python scripts (available at https://github.com/johannessonlab/InvDeNovo), which scanned MUMmer coordinate files to identify pairs of contigs in the de novo assemblies that aligned next to each other in the same chromosome of a reference assembly at two different locations and in different directions. If an inversion is present in the de novo assembly, this is the alignment pattern that is expected around the two inversion breakpoints (Supplementary Fig. 3). For each strain where candidate inversions larger than 100 kbp were identified, Illumina reads were then mapped to the de novo assembly using BWA^61^ in order to ensure that the signal of inversion was not caused by errors in the assembly procedure. Each breakpoint was also manually inspected to ensure that the signal was also not caused by a misalignment to repetitive sequences.

### Confirmation of inversion breakpoints

Inversions detected in this study from de novo Illumina assemblies, and not verified by other sequencing data, were verified by using PCR and Sanger sequencing. DNA from strains CJ01, CJ02, 7585, 7586 and CJ24 was obtained by incubating conidia or mycelial tissue in 10% Chelex^®^ 100 (Bio-Rad Laboratories) for 20 min at 95 °C. Primer pairs were designed around the putative inversion breakpoints (Supplementary Fig. 2, Supplementary Table 6). For the inversions in CJ01 and 7585, primers were designed to also match the nuclei of the alternative mating-type homokaryon from the same heterokaryons (i.e., CJ02 and 7586, respectively) enabling different primer combinations to confirm inverted or collinear gene order. In some cases, where breakpoints were found in non-aligning regions (7585 breakpoint 2, CJ24 breakpoint 4), it was impossible to determine the exact location of the breakpoint. Two primer pairs, one at each end of the region that did not align, were used to confirm presence of these breakpoints. PCRs were performed for each breakpoint and homokaryon pair simultaneously, for both inversion and collinear primer combinations. The Phusion^®^ High Fidelity PCR kit (Thermo Scientific) was used for 10 µl reactions with the following thermal cycler conditions: 98 °C (30 s) [98 °C (10 s). *°C (30 s), 72 °C (1 min)]×30, 72 °C (10 min), where * depended on primer combination. PCR products were sequenced with the BigDye Terminator v3.1 cycle sequencing kit (Thermo Fisher) and read on an ABI3730xl DNA Analyzer (Applied Biosystems).

### Filtering and enrichment analysis of inversions

Only inversions where both breakpoints were identified were included in this study. For inversions larger than 100 kbp, we verified breakpoints using PCR and Sanger sequencing and included only those that could be identified from at least two different types of data (i.e., both Illumina and PacBio, or both Illumina and PCR/Sanger). Inversions smaller than 100 kbp were included in the analysis only if they were found in at least two different strains. Supplementary Table 5 lists all inversions remaining after the final filtering, and Supplementary Table 4 lists all inversions larger than 100 kbp, where extra supporting evidence is available.

Enrichment of inversions in the non-recombining region on the mating-type chromosome were calculated by comparing the number of inversions per base-pair in the region of suppressed recombination on the mating-type chromosome (8,320,000 bp in strain 2509a, based on its maximum possible size as given by Corcoran et al.^27^) to the rest of the genome (30,097,195 bp in strain 2509a) using a one-sided binomial test. If the same inversion was found in several strains it was only counted once and the clusters of overlapping inversions found on the *mat* chromosome of Lineages 5 and 6 and on chromosome 5 on four Lineage 10 strains were also only counted as single inversion events.

### Data availability

Raw PacBio reads generated in this study have been deposited in the Sequence Read Archive as BioProject PRJNA398702, and raw Illumina mate-pair reads as BioProject PRJNA239947. Genome assemblies have been deposited at Figshare^62,63^. Scripts for identifying inversions from fragmented assemblies is available through GitHub: https://github.com/johannessonlab/InvDeNovo.

## Electronic supplementary material

Supplementary Information
