# Supplementary material for: Large-scale suppression of recombination predates genomic rearrangements in Neurospora tetrasperma
Source: Nat Commun. 2017 Oct 26;8:1140. doi: 10.1038/s41467-017-01317-6 (PMC5658415; doi:10.1038/s41467-017-01317-6)
Supplement: Supplementary file 1 — Supplementary Information [file 41467_2017_1317_MOESM1_ESM.pdf]

Supplementary Material

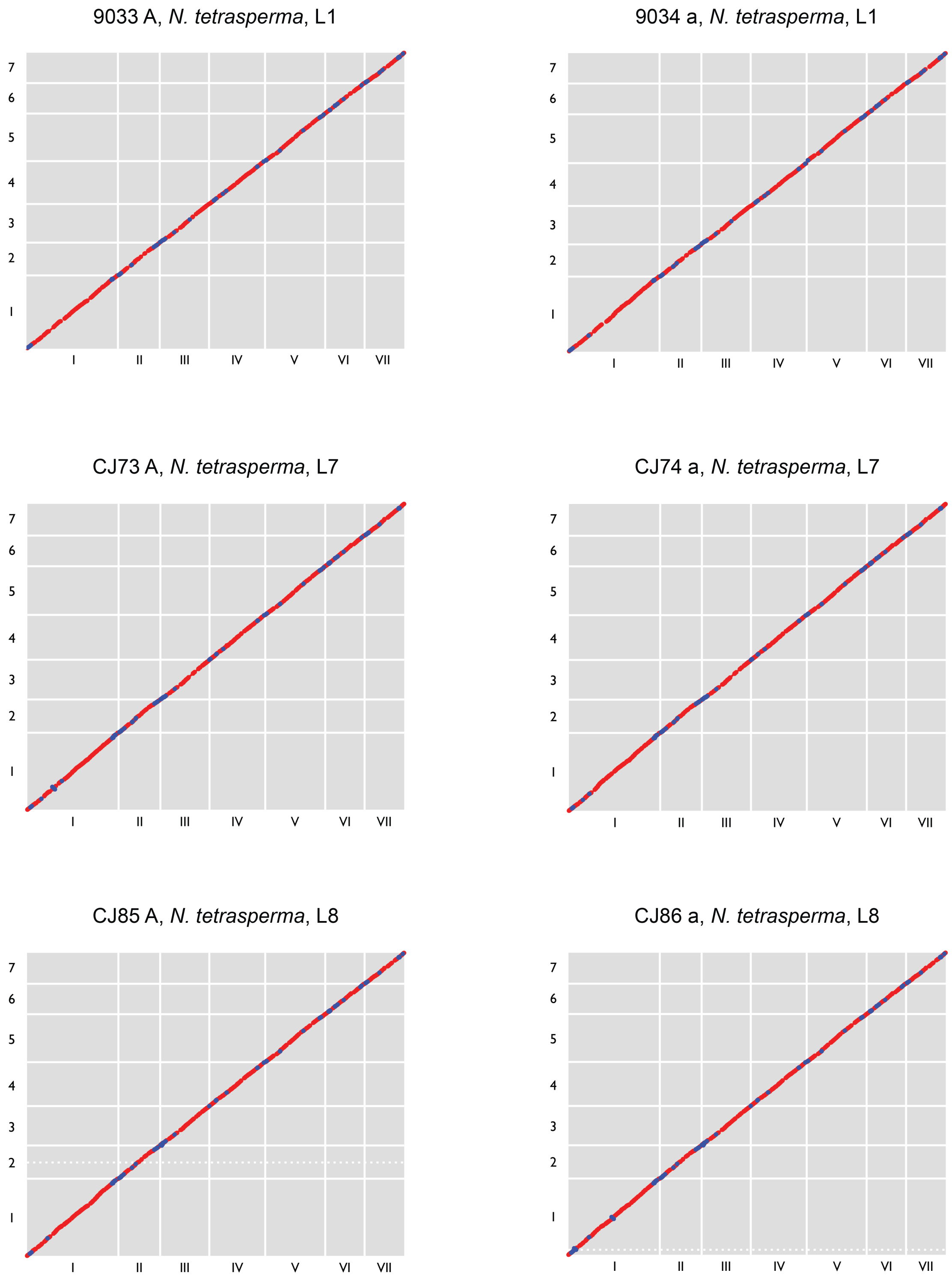

Supplementary Figure 1

Supplementary Figure 1 (continued)

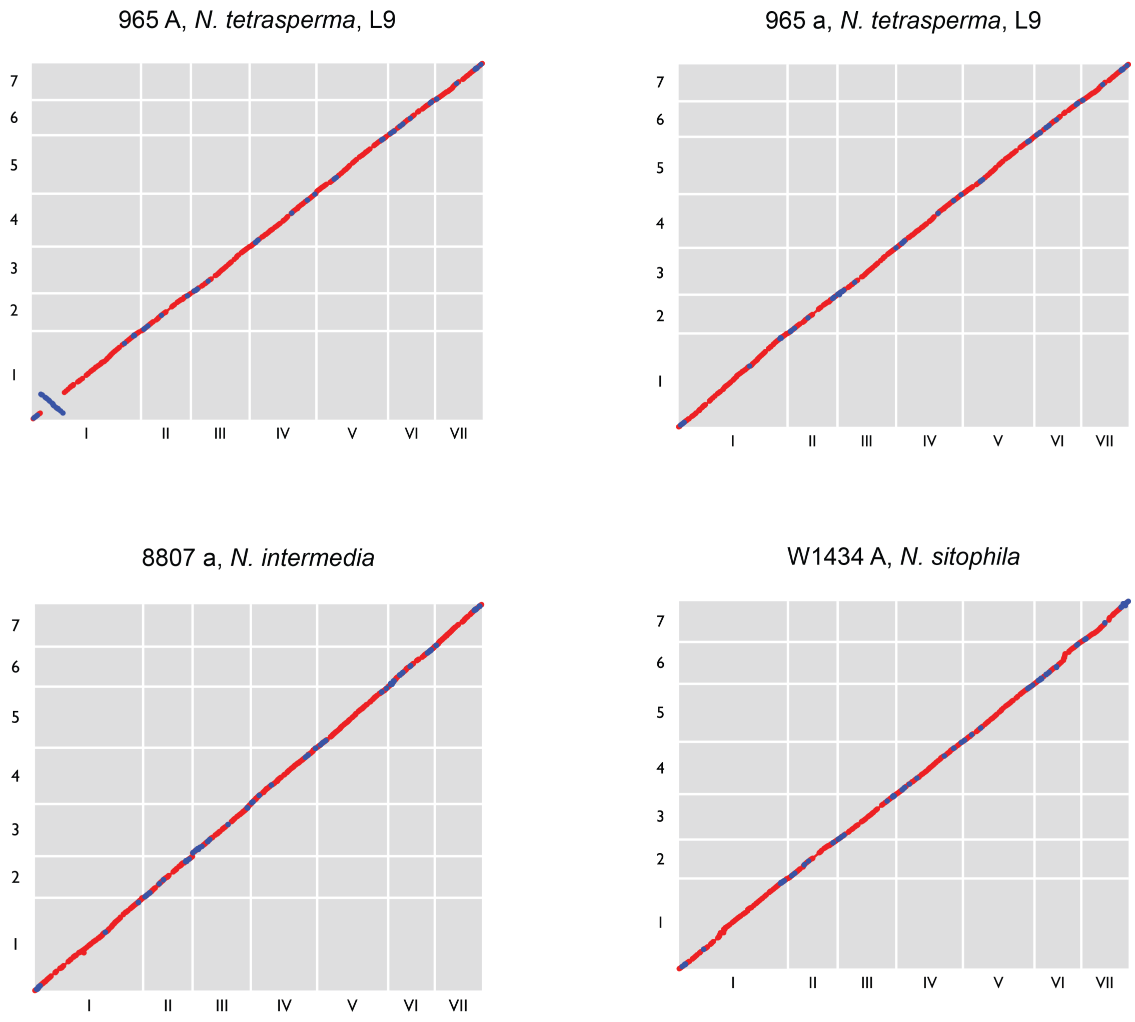

Supplementary Figure 1. Dotplot showing whole genome pairwise alignments between all strains sequenced for this study and the *N. crassa* OR74A assembly.

The seven chromosomes of *N. crassa* (I-VII) are shown at the bottom of each plot and the corresponding chromosomes of the sequenced strains (1-7) are shown on the left. Red dots signify collinear alignment blocks and blue dots signify inverted. A number of smaller inversions are found in all strains, but the majority are smaller than 20 kbp and large scale gene order is well conserved between the sequenced strains. Even *N. intermedia* 8807, which is estimated to have diverged from the other strains ~3 MYA[1], does not show an elevated number of inversions.

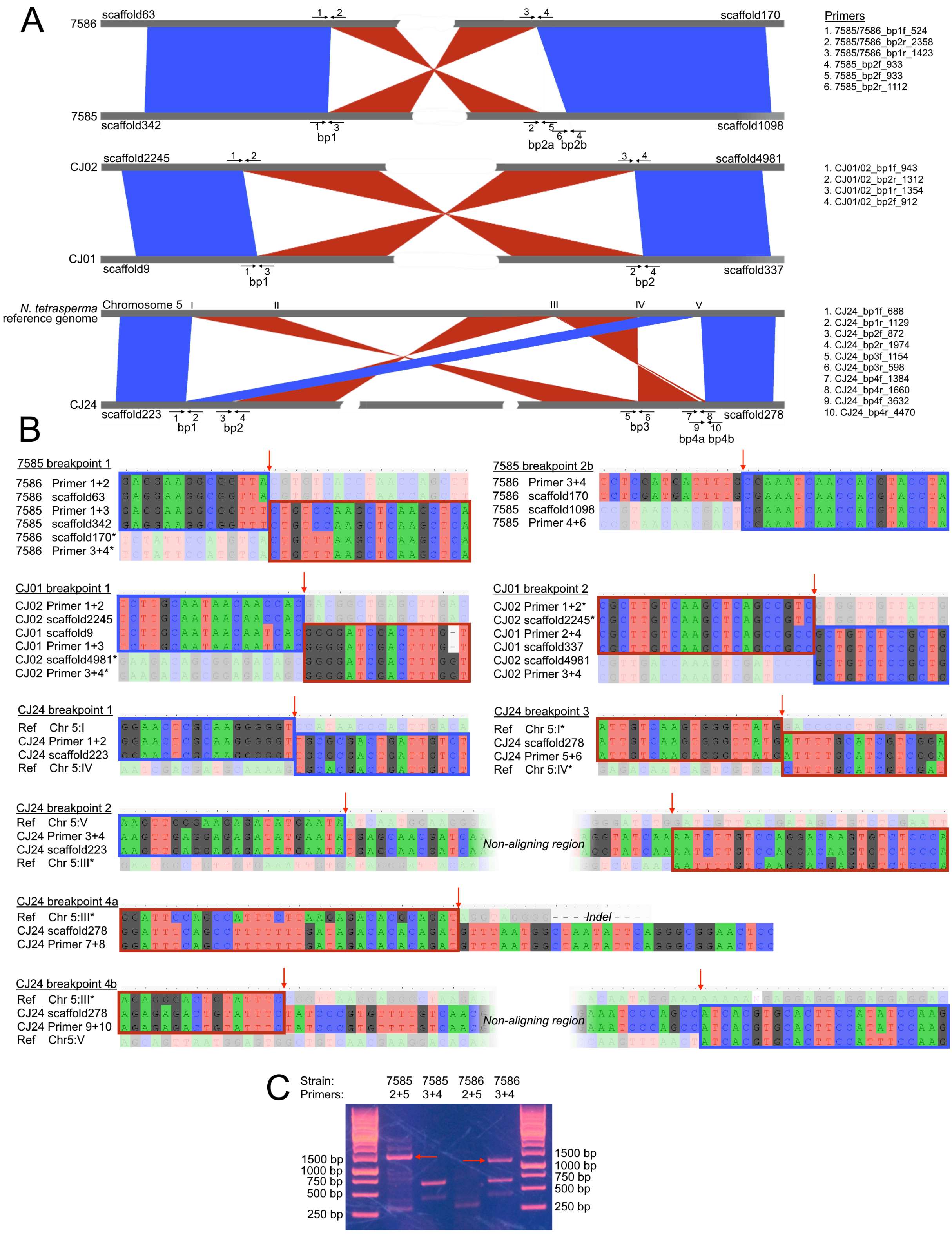

Supplementary Figure 2. Verification of inversions detected from Illumina data through PCR and Sanger sequencing.

(A) Map of inversions in 7585, CJ01 and CJ24 with approximate primer binding sites, targeting inversion breakpoints. Not drawn to scale. **(B)** Multiple sequence alignments of

genomic scaffolds and Sanger sequences across inversion breakpoints. Blue boxes represent collinear alignments and red boxes inverted alignments compared to reverse complemented sequences (*). Breakpoints are marked by an arrow. CJ24 breakpoints 2 and 4b contain regions up to a few hundred bp that do not align to the reference genome, making the exact breakpoint location impossible to pinpoint. These regions have been contracted in the figure, flanked by red arrows. **(C)** Verification of 7585 breakpoint 2a through PCR, because of sequencing complications. Expected product lengths can be obtained by subtracting the primer binding site coordinates (given at the end of primer ID's) from each other. Products contain some misprimed amplicons. Nevertheless, the same primer combinations caused the same misprimed amplicons regardless of strain, and unique bands of expected sizes were also obtained (marked by red arrows).

A

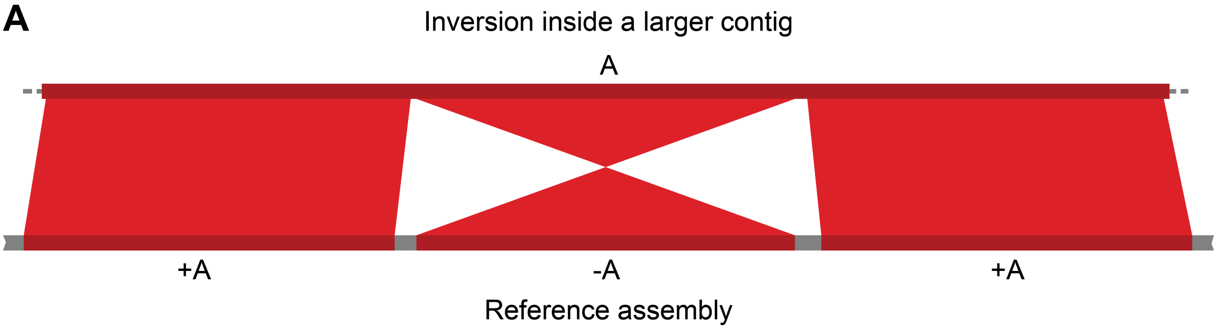

B

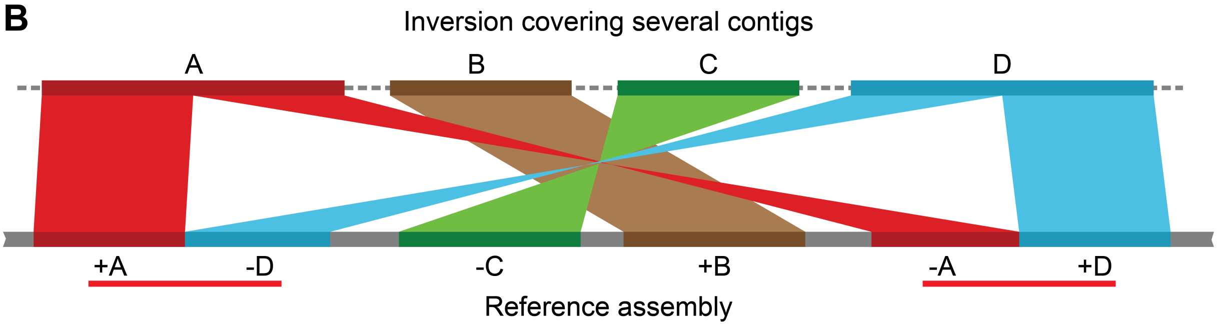

Supplementary Figure 3. Schematic model of method used for identifying inversions from fragmented *de novo* assemblies.

Inversions can be found by aligning two genome assemblies to each other, even if one of them is a highly fragmented *de novo* assembly. **(A)** If an inversion is located entirety within a contig of one assembly, it is possible to identify it as a block that aligns in the opposite direction compared to the rest of the contig (here marked as -A instead of +A) when aligned to the other assembly. **(B)** When inversions span several contigs, finding the exact size is more difficult. But if both inversion breakpoints are located within two different contigs, it will produce a specific alignment pattern that can be identified. In the figure, we show a fragmented assembly consisting of contigs A-D and carrying an inversion starting within contig A and ending within contig D. When aligned to a non-fragmented assembly, contig A and D will be aligned next to each other at both inversion breakpoints (red underlined regions). Contig A will be aligned adjacent to the 5'-end of D at both breakpoints with switched alignment direction (here shown with + or -) compared to the other breakpoint. Since a *de novo* genome assembler will produce contigs assembled in a random direction, A and D can share the same direction at one breakpoint (for example +A,+D), but they will then both be aligned in the other direction at the other breakpoint (-A,-D). For the same reason, the alignment direction of any contigs placed between A and D will be uninformative when identifying inversions.

Supplementary Table 1. Optical Mapping scaffold placement statistics and data availibility for three *N. tetrasperma* genomes.[1]

Strain	Num placed contigs	Total size placed contigs (Mbp)	Genome covered (%)	Average gap size (Kbp)	Physical depth	SRA accessions [2]
9033	85	35.0	90.0	41.6	~321 X	SRX485184<br>SRX485178
9034	98	34.2	86.1	47.2	~90 X	SRX485185<br>SRX485178
965A	51	36.7	91.9	41.0	~380 X	SRX485186<br>SRX485182

[1] The completed genome assemblies are available at https://dx.doi.org/10.6084/m9.figshare.5327515.v1[2].

[2] Sequence Read Archive accessions for mate-pair and paired end Illumina HiSeq reads.

Supplementary Table 2. PacBio sequencing assembly statistics and data availibility for five sequenced *N. tetrasperma* genomes and one each of *N. sitophila* and *N. intermedia*.[1]

Strain	Species	Number of unitigs	Number of placed unitigs[2]	Assembly size (Mbp)	Mean subread length	Mean read depth
CJ85	*N. tetrasperma*	29	8	38.5	6273	89.9 X
CJ86	*N. tetrasperma*	37	8	38.6	6996	88.0 X
CJ73	*N. tetrasperma*	27	7	39.0	6996	74.8 X
CJ74	*N. tetrasperma*	16	7	38.9	7102	63.7 X
965a	*N. tetrasperma*	24	7	38.6	6446	59.1 X
W1434	*N. sitophila*	15	7	39.2	7460	80.2 X
8807	*N. intermedia*	14	7	41.3	6884	69.3 X

[1] The completed genome assemblies are available at https://dx.doi.org/10.6084/m9.figshare.5327515.v1[2]. Raw PacBio reads have been deposited in the Sequence Read Archive as BioProject PRJNA398702.

[2] Number of unitigs that align to the seven chromosomes of the *N. crassa* OR74 reference assembly[3].

Supplementary Table 3. *De novo* assembly statistics for the 92 *N. tetrasperma* strains from Corcoran et al, 2016[4].[1]

	Assembly statistics							
Strain	Mating type	Total # contigs	Total length (Mbp)	N50 (kbp)	N90 (kbp)	Max length (kbp)	Coverage	Genomic GC (%)
7585	A	1155	36.7	105.1	20.98	488.4	~30 X	50.7
7586	a	1207	36.4	86.25	19.66	415.2	~30 X	50.8
9033	A	297	37.3	608.7	141.8	1739	~30 X	50.7
9034	a	333	38.1	598.6	126.9	1518	~50 X	50.8
965A	A	242	38.1	1141	358.9	2403	~50 X	50.1
965a	a	391	37.9	496.0	121.6	2049	~30 X	50.8
CJ01	A	718	37.9	193.3	51.47	943.9	~30 X	49.9
CJ02	a	18421	42.7	101.2	0.954	1261	~30 X	49.8
CJ03	A	754	37.9	188.9	46.51	748.7	~30 X	49.9
CJ04	a	2144	37.6	163.9	25.23	831.1	~30 X	49.9
CJ05	A	622	37.9	217.2	63.86	911.1	~30 X	49.9
CJ06	a	606	37.9	252.9	73.21	945.2	~30 X	49.9
CJ07	A	6849	39.9	204.0	9.591	1101	~30 X	49.6
CJ08	a	650	37.8	217.5	62.36	1090	~30 X	49.9
CJ09	A	721	37.9	226.3	53.16	631.8	~30 X	49.8
CJ10	a	628	37.8	237.8	70.49	870.5	~30 X	49.9
CJ11	A	4426	39.2	177.5	38.77	682.1	~30 X	49.7
CJ12	a	4028	39.0	178.0	31.06	587.9	~30 X	49.7
CJ13	A	621	38.0	236.1	73.73	1089	~30 X	49.8
CJ14	a	770	37.9	224.9	70.48	1103	~30 X	50.0

CJ15	A	584	37.9	232.9	70.13	873.5	~30 X	49.9
CJ16	a	633	37.9	230.9	69.06	766.7	~30 X	49.9
CJ17	A	34302	47.8	23.66	0.538	852.2	~30 X	49.4
CJ18	a	22969	44.9	80.80	0.681	851.4	~30 X	49.4
CJ19	A	641	38.0	219.0	55.40	765.7	~30 X	49.8
CJ20	a	603	37.7	216.5	68.77	765.0	~30 X	50.0
CJ21	A	11166	41.0	138.4	2.607	944.1	~30 X	49.7
CJ22	a	1699	38.0	200.0	53.06	1146	~30 X	50.0
CJ23	A	1438	38.1	159.7	30.36	595.0	~30 X	50.0
CJ24	a	1023	38.1	206.6	58.95	1083	~30 X	49.9
CJ25	A	640	39.0	211.7	62.80	1277	~30 X	49.3
CJ26	a	749	38.5	177.8	53.76	1278	~30 X	49.6
CJ27	A	988	39.1	172.4	46.78	842.2	~30 X	49.3
CJ28	a	775	38.6	172.5	58.96	885.3	~30 X	49.5
CJ29	A	972	38.9	163.7	45.08	1149	~30 X	49.4
CJ30	a	731	38.5	192.6	50.89	1066	~30 X	49.6
CJ31	A	1111	39.2	224.4	66.76	941.1	~30 X	49.2
CJ32	a	1610	38.6	196.4	53.81	874.2	~30 X	49.5
CJ33	A	1072	39.1	155.7	40.97	684.3	~30 X	49.3
CJ34	a	1003	38.7	149.4	43.21	569.7	~30 X	49.6
CJ35	A	1566	38.3	65.19	18.42	235.2	~30 X	49.6
CJ36	a	1259	38.5	139.9	34.80	805.0	~30 X	49.7
CJ37	A	703	39.4	190.7	60.07	784.5	~30 X	49.1
CJ38	a	711	38.6	177.8	68.38	795.5	~30 X	49.6
CJ39	A	668	39.1	254.6	66.56	778.2	~30 X	49.2

CJ40	a	759	38.5	187.8	54.20	766.3	~30 X	49.6
CJ41	A	2179	38.8	56.18	14.53	294.0	~30 X	49.5
CJ42	a	751	38.6	181.9	56.64	863.0	~30 X	49.5
CJ43	A	2388	38.7	81.48	17.44	320.5	~30 X	49.7
CJ44	a	1270	38.5	141.8	37.37	472.8	~30 X	49.7
CJ45	A	2387	38.7	49.45	12.92	235.8	~30 X	49.5
CJ46	a	725	38.5	203.9	62.48	941.5	~30 X	49.6
CJ47	A	768	39.1	182.3	54.09	917.8	~30 X	49.2
CJ48	a	913	39.1	194.6	56.22	959.9	~30 X	49.2
CJ49	A	791	39.5	194.4	64.85	1048	~30 X	49.1
CJ50	a	1064	38.5	178.3	53.95	770.1	~30 X	49.4
CJ51	A	3126	38.2	50.19	10.30	249.0	~30 X	49.9
CJ52	a	16060	43.7	109.1	1.150	601.4	~30 X	49.3
CJ55	A	2523	37.5	97.87	18.19	369.9	~40 X	50.4
CJ56	a	2369	37.9	109.1	18.19	556.6	~40 X	50.2
CJ57	A	1856	37.9	123.7	23.01	562.2	~40 X	50.4
CJ58	a	1721	38.0	121.4	22.33	441.6	~30 X	50.3
CJ59	A	1613	37.9	121.5	24.82	495.6	~40 X	50.3
CJ60	a	2023	37.7	113.6	21.23	444.1	~40 X	50.4
CJ61	A	1632	38.1	112.3	24.24	528.6	~30 X	50.2
CJ62	a	1290	38.1	133.1	29.32	670.4	~40 X	50.2
CJ63	A	1806	38.0	117.0	21.88	613.2	~40 X	50.3
CJ64	a	1374	38.2	141.5	27.84	677.7	~40 X	50.2
CJ65	A	1445	37.9	125.0	28.95	626.5	~40 X	50.0
CJ66	a	2262	37.9	106.3	19.56	556.8	~40 X	50.1

CJ67	A	2004	38.2	109.3	20.18	502.6	~40 X	50.2
CJ68	a	1660	38.1	122.3	23.18	674.1	~30 X	50.3
CJ69	A	2286	38.1	100.2	18.09	451.1	~40 X	50.1
CJ70	a	1602	38.6	107.5	26.68	514.7	~40 X	49.8
CJ71	A	2015	38.1	129.1	21.47	457.6	~40 X	50.3
CJ72	a	1377	38.2	131.5	29.23	669.5	~40 X	50.2
CJ73	A	2222	38.4	111.5	17.56	421.5	~30 X	50.0
CJ74	a	2103	38.5	101.5	20.04	415.8	~40 X	49.9
CJ75	A	1486	38.4	102.2	25.45	434.4	~40 X	50.1
CJ76	a	1464	38.2	134.5	27.43	669.3	~40 X	50.2
CJ77	A	1209	38.3	116.2	31.49	425.7	~40 X	50.1
CJ78	a	2599	36.3	92.69	21.49	325.6	~40 X	51.4
CJ79	A	2139	38.2	105.6	19.01	356.4	~40 X	50.4
CJ80	a	2805	36.7	81.81	18.60	468.4	~40 X	51.2
CJ81	A	1413	38.4	112.2	26.31	479.8	~40 X	49.9
CJ82	a	1622	38.5	110.5	25.69	392.7	~30 X	49.9
CJ83	A	2023	38.4	101.8	19.95	401.6	~30 X	50.1
CJ84	a	1166	38.6	142.5	36.23	591.3	~40 X	49.7
CJ85	A	2305	38.0	109.1	18.31	549.6	~40 X	50.4
CJ86	a	1605	38.0	119.2	24.33	524.8	~40 X	50.3
CJ87	A	17459	41.7	72.31	0.963	437.3	~30 X	50.3
CJ88	a	16892	42.3	71.21	1.045	437.8	~30 X	50.1

[1] The assembled genomes are available at https://dx.doi.org/10.6084/m9.figshare.5327809.v1[5].

Supplementary Table 4. Inverted regions larger than 100 kbp found in *N. tetrasperma*.

Lineage	Strains	Chr	Data[1]	Size (bp)	Start coordinate [2]	End coordinate [2]	Support[3]
L4	7585	1	Illumina	257,012	2,304,771	2,561,783	PCR
L5	All *mat A* strains in L5	1	Illumina	6,729,785	732,329	7,462,114	L6 reference genomes[4]
L7	All *mat A* strains in L7	1	PacBio	318,347	6,572,763	6,891,110	Illumina[5]
L8	All *mat a* strains in L8	1	PacBio	255,806	4,805,724	5,061,530	Illumina[5]
L9	965A	1	Optical mapping	1,941,612	6,772,348	8,713,960	Illumina
L10	CJ01	1	Illumina	3,632,717	4,332,431	7,965,148	PCR
L10	CJ21, CJ22, CJ23, CJ24	5	Illumina	417,226	96,060	513,286	PCR

[1] Type of data where inversion was first identified.

[2] Coordinates given for the breakpoint when aligned to the 2509 *mat a* reference genome.[6]

[3] Type of secondary data that supports the identified inversion.

[4] The pattern of inversion breakpoints that is found in all *mat A* strains in lineage 5 corresponds to the inversions that were identified in the lineage 6 2508 *mat A* strain sequenced by Ellison et al.[6]

[5] Only one breakpoint could be confirmed with Illumina, due to a large cluster of repetitive sequences surrounding the other. For the inversion in Lineage 7 the one breakpoint was found in all four *mat A* genomes and for the Lineage 8 inversion, it was found in all nine *mat a* genomes, giving us multiple assembly-independent datapoints.

Supplementary Table 5. Inversions larger than 1 kb [1] found in *N. tetrasperma*.

Chromosome	Start coordinate[2]	End coordinate[2]	Size (bp)	Number of hits[3]	Location[4]
1	279991	352979	72988	22	recombining
1	548863	556097	7234	57	recombining
1	732329	7462114	6729785 [5]	30	non-recombining
1	2304771	2561783	257012 [5]	1	non-recombining
1	4332431	7965148	3632717 [5]	1	non-recombining
1	4805724	5061530	255806 [5]	10	non-recombining
1	5738804	5755786	16982	2	non-recombining
1	6374901	6376114	1213	14	non-recombining
1	6572763	6891110	318347 [5]	4	non-recombining
1	6772348	8713960	1941612 [5]	1	non-recombining
1	7285213	7309212	23999	21	non-recombining
1	9003634	9022861	19227	5	recombining
1	9026355	9035333	8978	38	recombining
1	9084396	9094323	9927	38	recombining
1	9190232	9193161	2929	4	recombining
1	9291105	9294807	3702	6	recombining
2	95622	137829	42207	55	recombining
2	6040269	6052643	12374	32	recombining
3	76449	94467	18018	61	recombining
3	58482	105153	46671	26	recombining
3	682962	721398	38436	30	recombining
3	3121685	3128792	7107	3	recombining
3	3650653	3657421	6768	2	recombining
3	5632546	5640449	7903	41	recombining
4	2005613	2014291	8678	45	recombining
4	4357340	4401633	44293	7	recombining
4	4730416	4735395	4979	54	recombining
4	4734740	4749514	14774	9	recombining
4	4756352	4770430	14078	28	recombining
4	4864863	4882478	17615	8	recombining

5	96060	513286	417226 [5]	4	recombining
5	499226	514523	15297	49	recombining
5	535228	556550	21322	54	recombining
5	1833051	1842914	9863	44	recombining
5	3673570	3691824	18254	2	recombining
5	3833318	3872698	39380	18	recombining
6	123407	128291	4884	13	recombining
6	163494	213506	50012	2	recombining
6	261829	305573	43744	2	recombining
6	516684	532300	15616	56	recombining
6	606365	693014	86649	31	recombining
6	2182836	2184719	1883	36	recombining
6	2516527	2540516	23989	36	recombining
6	3549368	3596893	47525	8	recombining
6	3650045	3658661	8616	22	recombining
7	325569	351901	26332	42	recombining
7	1098423	1149434	51011	2	recombining
7	1111255	1149434	38179	61	recombining
7	3414311	3418916	4605	28	recombining
7	3494575	3523457	28882	10	recombining
7	3632500	3645415	12915	2	recombining

[1] Only inversions found in at least two different strains are included, with the exception of inversions larger than 100 kb shown in Supplementary Table 4.

[2] Start and end coordinates were defined as the last non-inverted base before the inverted region, and the first non-inverted base after.

[3] The number of strains that the inversion was identified by aligning to 2509 a reference genome. Only inversions found in at least two strains, or those verified by PCR or PacBio sequencing were included.

[4] Inversion located in the freely recombining region or in the non-recombining region.

[5] For further details of inversions larger than 100 kb, see Supplementary Table 3.

Supplementary Table 6. Primers used for PCR confirmation of newly identified inversions.

Primer ID	Target inversion	Sequence (5'–3')	T_m (°C)
7585/7586_bp1f_524	200 kb, breakpoint 1	CCATCATCCGCATCCAATAT	64.4
7585/7586_bp1r_1423	200 kb, breakpoint 1	GGGCTGCTACTGATTCTGCT	63.4
7585_bp2f_933	200 kb, breakpoint 2	ATAACAATATGGCCAGCCAG	61.3
7586_bp2f_334	200 kb, breakpoint 2	TGGATGATTGTCATCAGAGG	61.2
7585/7586_bp2r_2358	200 kb, breakpoint 2	AGACCAAGCTGGTTAGGTGA	61.3
7585_bp2r_1112	200 kb, breakpoint 2	AGAAGTCGTCGGTTCAGATG	61.2
CJ01_bp1f_943	4 Mb, breakpoint 1	GTGTCAACCTTGTACGACAA	56.9
CJ01_bp1r_1354	4 Mb, breakpoint 1	TCGTCTTGATATGACATCCC	59.9
CJ01_bp2f_912	4 Mb, breakpoint 2	TCGGATGAATTTGACAAGGA	63.0
CJ01_bp2r_1312	4 Mb, breakpoint 2	AGTCAGGGTCATGGCAACTT	63.5
CJ24_bp1f_688	400 kb, breakpoint 1	GGAGGCTGATACCGTTGAAC	62.7
CJ24_bp1r_1129	400 kb, breakpoint 1	CATTGTGCGCTTCATCCATA	63.8
CJ24_bp2f_872	400 kb, breakpoint 2	ATGGTTCAGGATCAGGTCAC	60.7
CJ24_bp2r_1974	400 kb, breakpoint 2	CGAAGACGAACAGCAACTCA	63.5
CJ24_bp3f_1154	400 kb, breakpoint 3	GTGTTCAGGCCCAAGCAGCT	68.1
CJ24_bp3r_598	400 kb, breakpoint 3	CACTTTGCATTGGATCGTGC	65.7
CJ24_bp4f_1384	400 kb, breakpoint 4.1	ACTGAGACGAAATGGCTTTG	61.2
CJ24_bp4r_1660	400 kb, breakpoint 4.1	GCGTATAAGTACCACAAGCAGC	61.8
CJ24_bp4f_3632	400 kb, breakpoint 4.2	GGCAATAGCCTTCATCATGT	60.8
CJ24_bp4r_4470	400 kb, breakpoint 4.2	CGCTGGCTTGGAAGTATATG	61.5

Supplementary References

1. Corcoran, P. *et al.* A global multilocus analysis of the model fungus Neurospora reveals a single recent origin of a novel genetic system. *Mol. Phylogenet. Evol.* **78,** 136–147 (2014)
2. Sun, Y., Corcoran, P., Svedberg, J. & Johannesson, H. De novo genome assemblies of *Neurospora tetrasperma* based on Illumina HiSeq reads. Figshare. https://dx.doi.org/10.6084/m9.figshare.5327809.v1 (2017)
3. Galagan, J. E. *et al.* The genome sequence of the filamentous fungus Neurospora crassa. *Nature* **422,** 859–868 (2003).
4. Corcoran, P. et al. Introgression maintains the genetic integrity of the mating-type determining chromosome of the fungus Neurospora tetrasperma. Genome Res. **26**, 486–498 (2016).
5. Sun, Y., Svedberg, J., Hiltunen, M., Corcoran, P. & Johannesson, H. High-quality *Neurospora tetrasperma* genome assemblies. Figshare. https://dx.doi.org/10.6084/m9.figshare.5327515.v1 (2017)
6. Ellison, C. E. *et al.* Massive changes in genome architecture accompany the transition to self-fertility in the filamentous fungus Neurospora tetrasperma. *Genetics* **189,** 55–69 (2011).
